# Multidrug-Resistant Tuberculosis in Patients with Chronic Obstructive Pulmonary Disease in China

**DOI:** 10.1371/journal.pone.0135205

**Published:** 2015-08-18

**Authors:** Jiang-nan Zhao, Xian-xin Zhang, Xiao-chun He, Guo-ru Yang, Xiao-qi Zhang, Wen-gen Xin, Huai-chen Li

**Affiliations:** 1 Department of Respiratory Medicine, Provincial Hospital Affiliated to Shandong University, Jinan, China; 2 Department of Respiratory Medicine, Shandong Provincial Chest Hospital, Jinan, China; 3 Department of Respiratory Medicine, Chest Specially Hospital of Weifang, Weifang, China; 4 Department of Tuberculosis Medicine, Chest Specially Hospital of Weifang, Weifang, China; Universidad Nacional de La Plata., ARGENTINA

## Abstract

**Background:**

Relatively little is known about the specific relationship and impact from chronic obstructive pulmonary disease (COPD) on multidrug-resistant tuberculsosis (MDR-TB).

**Methods:**

We conducted a retrospective study included patients aged ≥40 years with a confirmed pulmonary TB at three tertiary hospitals (Shandong, China) between January 2011 and October 2014. Univariable and multivariable analyses were performed to identify the relationship of MDR-TB and COPD.

**Results:**

A total of 2164 patients aged ≥ 40 years with available results of drug susceptibility test (DST) and medical records were screened for this study: 268 patients with discharge diagnosis of COPD and 1896 patients without COPD. Overall, 14.2% of patients with COPD and 8.5% patients without COPD were MDR-TB. The rate of MDR-TB were significantly higher in patients with COPD (P<0.05). Migrant (odds ratios (OR) 1.32, 95% confidence interval (CI) 1.02–1.72), previous anti-TB treatment (OR 4.58, 95% CI 1.69–12.42), cavity (OR 2.33, 95% CI 1.14–4.75), and GOLD stage (OR 1.86, 95% CI 1.01–2.93) were the independent predictors for MDR-TB among patients with COPD.

**Conclusions:**

MDR-TB occurs more frequently in patients with underlying COPD, especially those with being migrant, previous anti-TB therapy, cavity and severe airway obstruction.

## Introduction

The emergence and spread of multidrug-resistant tuberculosis (MDR-TB) is hampering efforts to control and manage the TB disease worldwide, since the effect of standard short course chemotherapy is less and second line drugs are less potent, more toxic and much more expensive [1,2]. Moreover, MDR-TB strains, are highly pathogenic, have the great potential for transmission and add to mortality incrementally [3,4]. The latest Global Tuberculosis Report indicates an estimated 5% of TB cases (3.5% of new and 20.5% of previously treated cases) had MDR-TB in 2013, which translates into a somber of 480,000 people developed MDR-TB [5]. According to the fifth national tuberculosis epidemiological survey in China, the rate of MDR-TB was 6.8% with an estimated 339,000 incident cases among population over 15 years old in 2010 [6].

As a consequence of population growth, fuel consumption and smoking habit, China is also witnessing an escalating epidemic of chronic obstructive pulmonary disease (COPD), of which the overall prevalence was 8.2% among all individuals 40 years of age or older [7]. Previous literature suggested that pulmonary TB was a strong risk factor of COPD [8–10]. Meanwhile, as evidenced by a number of studies, COPD is associated with a higher risk of developing active TB [11,12]. The double burden of disease is a serious challenge and poses a threat for China health systems.

COPD is a known risk factor for fluoroquinolone-resistant TB [13], however, there remains a paucity of convincing data about the relationship of resistance to other anti-TB drugs and COPD. Since that history of pulmonary TB is an independent risk factor for developing COPD and that prior treatment for TB is a risk factor for MDR-TB, it is assumed that the incidence of MDR is higher in pulmonary TB patients combined with COPD. The objectives of this study, therefore, is to determine the association between MDR-TB and COPD.

## Methods

### Ethics statement

The study was approved by the Ethic Committee of Shandong Provincial Hospital, affiliated to Shandong University (approval numbers: 2014–0009). Patients’ records were anonymized and de-identified prior to analysis.

### Setting, Study Population and data collection

Shandong Province held 97,894,300 permanent residents and 13,700,000 migrants from other provinces of China in 2014, covering a total area of 158,000 km^2^. This retrospective cohort study was conducted in Shandong Provincial Hospital, Shandong Provincial Chest Hospital and Chest Specially Hospital of Weifang, which elaborated a common research protocol. Shandong Provincial Hospital is a 2000-bed tertiary-care university-affiliated, comprehensive public hospital with the largest scale in Shandong province. Shandong Provincial Chest Hospital and Chest Specially Hospital of Weifang are two major TB referral hospitals in the Shandong province. Patients treated in three hospitals are either self-referred or referred from general hospitals and community clinics, as well as district TB prevention and treatment clinics in Shandong and other provinces of China.

In this study, consecutive patients were enrolled with a diagnosis of pulmonary TB from January 1, 2011 to October 31, 2014. According to the previous literature, COPD is prevalent and underrecognized in individuals aged ≥ 40 years in China [7]. Therefore, we only included patients 40 years of age or older. All patients with both DST results and medical records available were included for further analysis. Patients’ information were routinely collected and recorded by trained research coordinators over the entire study period. We obtained the following information from medical records for all study patients: age, sex, occupation, residence, smoking history, excess alcohol consumption, history of close contact with a TB patient, co-morbid conditions (according to the discharge diagnosis), laboratory data, chest radiography interpretation by board-certified radiologists, ever hospitalization in two years, hospital length-of-stay (LOS), duration of TB diagnostic delay (the onset of pulmonary symptoms to the diagnosis of TB) and hospital discharge status (survive, dead).

Patients with the combined discharge diagnosis of pulmonary TB and COPD were reviewed for the spirometry tests. COPD was diagnosed according to the guidelines of Global Initiative for Chronic Obstructive Lung Disease (GOLD) [14]. COPD was defined by a post-bronchodilator pulmonary function (forced expired volume in one second (FEV1)/forced vital capacity (FVC) ratio) of less than 70%, with prescription of a combination of various bronchodilators and anticholinergic agents. In our study, all COPD patients had been diagnosed before TB diagnosis.

The exclusion criteria included a diagnosis of bronchiectasis, interstitial lung disease, asthma, pulmonary edema, a recent history of myocardial infarction, left heart failure and pulmonary embolism, malignant tumor, a history of thoracotomy with pulmonary resection, or other disease that could potentially affect the spirometry test. Patients infected by HIV were also not included in our study, since HIV-positive patients will be transferred to HIV specialized hospital immediately in China.

### Drug susceptibility testing

For sputum culture, samples are digested with 4% sodium hydroxide for 15 min and then inoculated to acid-buffer Lowenstein-Jensen (L-J) media. The L-J culture media was incubated at 37°C. The growth situation of cultured TB was observed on the third and seventh days, subsequently once a week at least 8 weeks. DST was performed after subculturing with following concentrations of the drugs [15]: isoniazid (0.2 μg/mL), rifampicin (40 μg/mL), ethambutol (2.0 μg/mL), streptomycin (4.0 μg/mL), kanamycin (30 μg/mL), capreomycin (40 μg/mL), ofloxacin (2.0 μg/mL), para-aminosalicylic acid (1.0 μg/mL). The isolates were considered to be resistant if there was more than 1% growth on medium containing anti-TB drugs as compared with the growth on drug-free medium. External quality assessment (EQA) was conducted regularly by TB National Reference Laboratory.

### Definitions

Previously treated cases were defined as TB patients who had been receiving TB treatment for at least 30 days or who had documented evidence of prior treatment from the case report or surveillance database [16]. Retreatment categories included treatment default, treatment failure and relapse.

### Statistical analysis

Categorical variables were summarized as proportions; continuous variables were summarized with mean and standard deviation (SD). In univariable analysis, chi-squared test and Wilcoxon rank sum test were used to compare categorical variables, and Student’s t test was used to compare continuous variables. To identify independent factors that were associated with MDR-TB, univariable comparison and subsequent multivariable logistic regression analysis were used. The odds ratios (OR) and the 95% confidence interval (CI) were obtained using a logistic regression model, and P < 0.05 is considered to be statistically significant. To assess the discriminatory ability of the model, we calculated the c statistic, which represents the area under the receiver operating characteristic (ROC) curve, ranges from 0.5 (which indicates no better discrimination than chance) to 1.0 (perfect discrimination). Statistical analysis was performed using SPSS software, version 16.0.

## Results

### Demographic and clinical characteristics in patients with COPD

A total of 2164 patients aged ≥ 40 years with available results of DST and medical records were screened for this study: 268 patients with COPD and 1896 patients without COPD. Demographic and clinical characteristics of patients were recorded in Table 1. The mean age of COPD patients was 63 (mean±SD, 62.9±19.5) years and 84.7% were male. A significantly greater proportion of patients with COPD were male, current or former smokers and had previous anti-TB treatment (P<0.05). Patients with COPD had longer duration of TB diagnostic delay (days) (46.4±32.8 vs. 30.4±21.5; P = 0.037) and longer hospital LOS (days) (60.28±20.49 vs. 45.70±17.34; P = 0.002).

**Table 1:** Demographic/clinical characteristics and prevalence of anti-TB drug resistance of study populations.

	Patients without COPD	Patients with COPD	P value
	N = 1896 (%)	N = 268 (%)	
Sex			0.029
Male	1494(78.8)	227(84.7)	
Female	402(21.2)	41(15.3)	
Age	61.4±12.2	63.4±11.0	0.079
Occupation			
Others	528(27.8)	84(31.1)	0.246
Carder	68(3.6)	5(1.9)	0.153
Worker	388(20.5)	43(16.0)	0.102
Farmer	911(48.0)	136(50.7)	0.433
Residence			0.845
Rural	992(52.3)	138(51.5)	
Urban	904(47.7)	130(48.5)	
Migrant	322(17.0)	53(19.8)	0.263
BMI	19.1±3.4	19.9±3.3	0.592
Excess alcohol consumption^a^	388(20.5)	66(24.6)	0.128
Current or former Smokers	908(47.8)	218(81.3)	<0.001
TB contact^b^	138(7.3)	26(9.7)	0.174
Re-treatment case	484(25.5)	86(32.1)	0.026
Default	174(9.2)	24(9.0)	0.911
Failure	184(9.7)	22(8.2)	0.442
Relapse	126(6.6)	40(14.9)	<0.001
*Chest radiology*			
Cavity	1346(71.0)	176(65.7)	0.086
*Comorbidities *			
Hepatic cirrhosis	6(0.3)	2(0.7)	0.260
Hypoalbuminemia	964(50.8)	134(50.0)	0.845
Chronic renal failure	62(3.3)	8(3.0)	0.858
Cardio-cerebrovascular disease	104(5.5)	14(5.2)	0.888
Hypertension	226(11.9)	28(10.4)	0.543
Diabetes	946(49.9)	148(55.2)	0.103
Gastric ulcer	54(2.8)	12(4.5)	0.180
Combined extra-pulmonary TB	340(17.9)	58(21.6)	0.152
Duration of TB diagnostic delay(days)	30.4±21.5	46.4±32.8	0.037
Ever hospitalization in two years^c^	104(5.5)	44(16.4)	<0.001
Hospital LOS, days (mean ± SD)	45.7±17.3	60.3±20.5	0.002
Hospital mortality	6(0.3)	3(1.1)	0.089
Resistance to drugs			
Any first-line drug	480(25.3)	80(29.9)	0.118
Isoniazid	266(14.0)	52(19.4)	0.022
Rifampin	244(12.9)	48(17.9)	0.028
Isoniazid or rifampin (but not both)	184(9.7)	24(9.0)	0.741
MDR	162(8.5)	38(14.2)	0.004
Ethambutol	56(3.0)	8(3.0)	1.000
Streptomycin	280(14.8)	38(14.2)	0.854
Kanamycin	60(3.2)	16(6.0)	0.023
Capreomycin	72(3.8)	14(5.2)	0.314
Ofloxacin	208(11.0)	44(16.4)	0.011
MDR plus resistance to ofloxacin	68(3.5)	18(6.7)	0.043
MDR plus resistance to SLID	48(2.5)	8(3.0)	0.679
XDR	40(2.1)	8(3.0)	0.372
Para-aminosalicylic acid	56(3.0)	6(2.2)	0.567

Abbreviation: COPD, chronic obstructive pulmonary disease; BMI, body mass index; LOS, length of stay; MDR, multidrug-resistant; SLID, second line injectable drugs (including kanamycin and capreomycin); XDR, extensively drug-resistant

Note:

^a^ Excess alcohol consumption means more than 2 standard alcohol beverages per day.

^b^ TB contact was defined as a household member or colleague with TB.

^c^ Ever hospitalised in two years was due to an exacerbation of COPD.

Among 268 patients with COPD, 86 patients had previous anti-TB treatment. Compared to patients without COPD, the rate of retreatment in patients with COPD was significantly higher (25.5% vs. 32.1%; P = 0.026). Among 86 patients with COPD who had previous anti-TB treatment, 24(9.0%), 22(8.2%) and 40(14.9%) patients fell into the categories of treatment default, treatment failure and relapse, respectively. There was a greater percentage of relapse in the patients with COPD (14.9%) as compared to the those without COPD (6.6%) (P<0.001).

### Drug-resistant tuberculosis in patients with COPD

**Fig 1:**
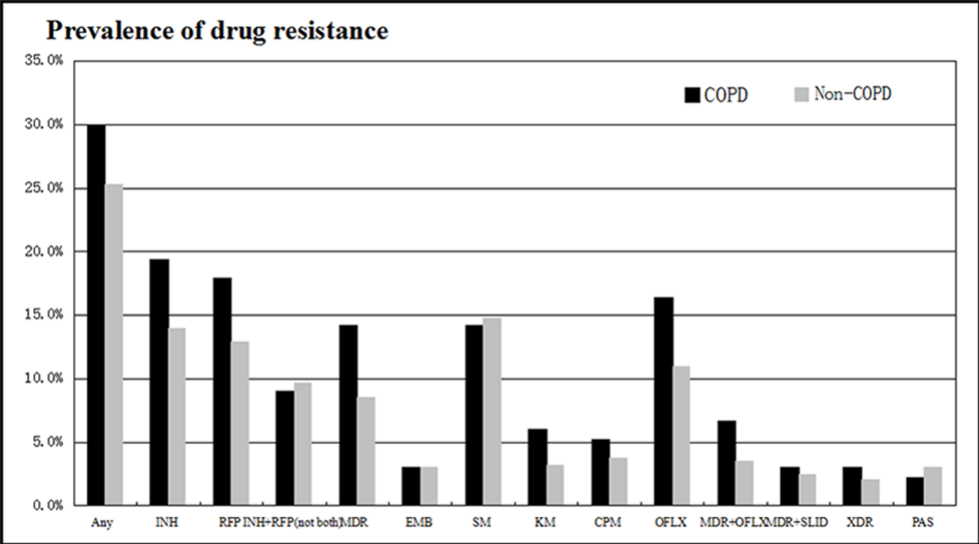
Overall drug resistance (%) in TB patients with and without COPD. (INH, isoniazid; RFP, rifampicin; MDR, multidrug-resistant; EMB, ethambutol; SM, streptomycin; KM, kanamycin; CPM, capreomycin; OFLX, ofloxacin; MDR+OFLX, MDR plus resistance to ofloxacin; MDR+SLID, MDR plus resistance to second line injectable drugs; XDR, extensively drug-resistant; PAS, para-aminosalicylic acid).

In our study, 29.9% of patients with COPD were resistant to at least one of the four first-line anti-TB drugs (isoniazid, rifampin, ethambutol, and streptomycin). The prevalence of drug resistance in COPD patients to isoniazid and rifampin was 19.4% and 17.9%, respectively. Overall, 14.2% of patients with COPD and 8.5% patients without COPD were MDR-TB. The rate of resistance to isoniazid, rifampicin, and MDR-TB were significantly higher in patients with COPD (P<0.05). For kanamycin, ofloxacin, and MDR plus resistance to ofloxacin, the proportion of patients with drug-resistant were significantly higher in COPD group (P<0.05). Among all patients with and without COPD, 3.0% and 2.1%, respectively, had extensively drug-resistant TB (XDR-TB). The detail rate of drug resistance in COPD patients were shown in Table 1. The percentage of resistance to the individual agents in patients with COPD compared to those without COPD were reflected by Fig 1.

### Risk factors of MDR-TB in patients with COPD

**Fig 2:**
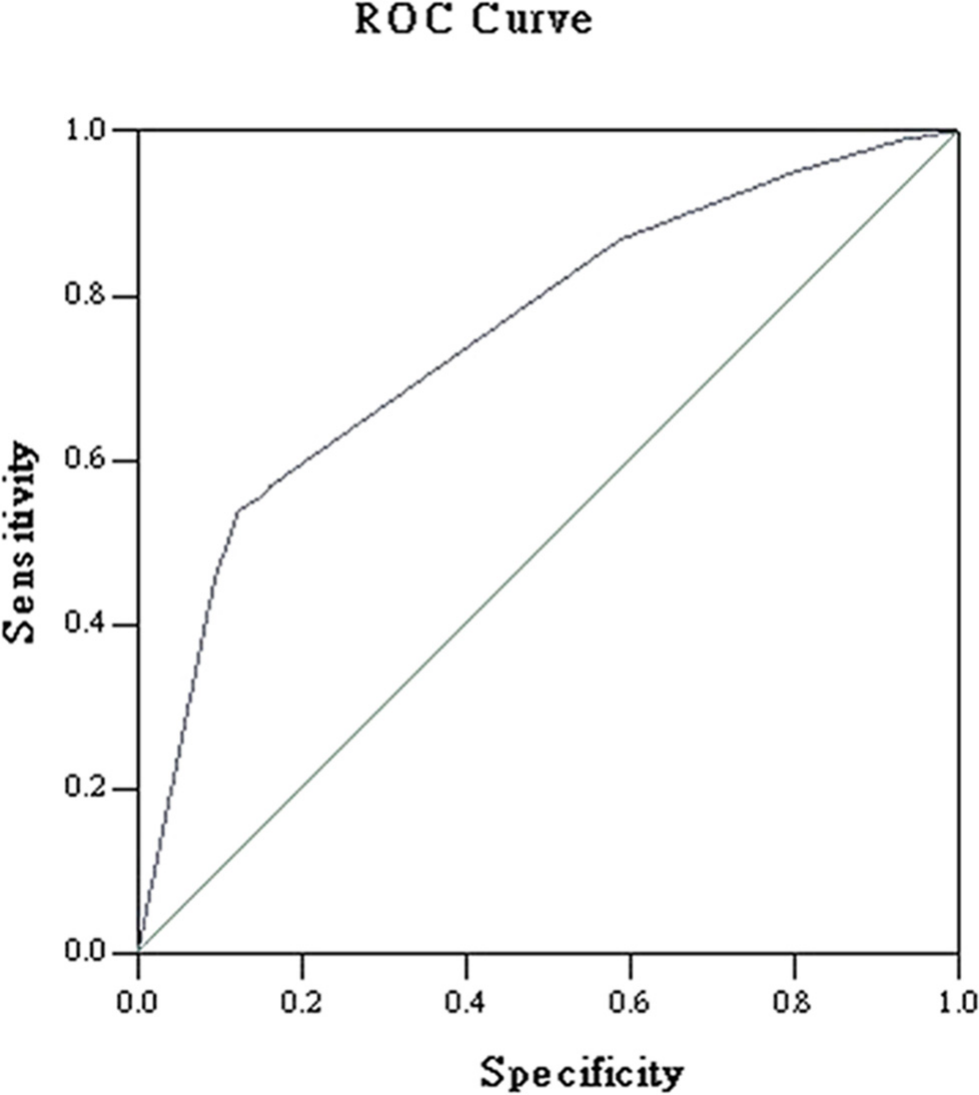
ROC curve to assess the discriminatory ability of the model predicting MDR-TB in patients with COPD.

Table 2 shows the results of the risk factors for MDR-TB in patients with COPD. Univariable comparison showed that the following characteristics predispose the presence of MDR-TB: migrant, current or former smokers, previous anti-TB treatment, cavity, hypoalbuminemia, GOLD stage, and ever hospitalization in two years. On the basis of the clinical variables included in univariable comparison, the final multiple logistic regression model predicting MDR-TB in patients with COPD were migrant (OR 1.32, 95% CI 1.02–1.72), previous anti-TB treatment (OR 4.58, 95% CI 1.69–12.42), cavity (OR 2.33, 95% CI 1.14–4.75), and GOLD stage (OR 1.86, 95% CI 1.01–2.93). The ROC curve is shown in Fig 2. The AUC was 0.80 (95%CI 0.75–0.84, P<0.001). The c statistic value, which represents by the AUC, is considered acceptable.

**Table 2:** Univariable and multivariable analysis of risk factors for MDR-TB in patients with COPD.

	Non-MDR-TB	MDR-TB	Univariable analysis		Multivariable analysis	
Variables	N = 230	N = 38	OR (95% CI)	P value	OR (95% CI)	P value
Sex						
Male	195(84.8)	32(84.2)	Reference			
Female	35(15.2)	6(15.8)	1.05(0.41–2.68)	1.000		
Age	62.9±13.9	59.2±19.2		0.088		
Occupation						
Others	70(30.4)	14(36.8)	1.33(0.65–2.73)	0.453		
Carder	4(1.7)	1(2.6)	1.53(0.17–14.04)	0.537		
Worker	36(15.7)	7(18.4)	1.22(0.50–2.98)	0.812		
Farmer	120(52.2)	16(42.1)	0.67(0.33–1.33)	0.295		
Residence						
Rural	120(52.2)	18(47.4)	Reference			
Urban	110(47.8)	20(52.6)	1.21(0.61–2.41)	0.604		
Migrant	37(16.1)	16(41.0)	3.63(1.75–7.52)	0.001	1.32(1.02–1.72)	0.038
BMI	19.2±2.6	18.1±2.9	0.99(0.97–1.01)	0.346		
Excess alcohol consumption^a^	54(23.5)	12(31.6)	1.50(0.71–3.18)	0.311		
Current or former Smokers	182(79.1)	36(94.7)	4.75(1.10–20.42)	0.023		
TB contact^b^	24(10.4)	2(5.3)	0.48(0.11–2.11)	0.552		
Re-treatment case	64(27.8)	22(57.9)	3.57(1.76–7.22)	<0.001	4.58(1.69–12.42)	0.003
*Chest radiology*						
Cavity	144(62.6)	32(84.2)	3.19(1.28–7.93)	0.009	2.33(1.14–4.75)	0.008
*Comorbidities *						
Hepatic cirrhosis	1(0.4)	1(2.6)	6.19(0.38–101.12)	0.264		
Hypoalbuminemia	108(47.0)	26(68.4)	2.45(1.18–5.09)	0.022		
Chronic renal failure	8(3.5)	0(0)	0.85(0.81–0.90)	0.606		
Cardio-cerebrovascular disease	10(4.3)	4(10.5)	2.59(0.77–8.72)	0.120		
Diabetes	122(53.0)	26(68.4)	1.92(0.92–3.99)	0.082		
Hypertension	22(9.6)	6(15.8)	1.77(0.67–4.71)	0.254		
Gastric ulcer	10(4.3)	2(5.3)	1.22(0.26–5.81)	1.000		
Combined extra-pulmonary TB	46(20.0)	12(31.6)	1.85(0.87–3.93)	0.135		
*COPD*, *GOLD stage* ^c^				0.016	1.86(1.01–2.93)	0.041
Mild	61(26.5)	4(10.5)				
Moderate	93(40.4)	16(42.1)				
Severe	64(20.0)	12(31.6)				
Very severe	12(5.2)	6(15.8)				
Inhalation corticosteroids treatment^d^	82(35.7)	14(36.8)	1.05(0.52–2.15)	1.000		
Duration of COPD (years)	8.32±7.93	9.29±8.43	0.99(0.98–1.01)	0.849		
Duration of TB diagnostic delay (days)	46.8±31.9	50.3±27.2	1.10(0.67–1.82)	0.355		
Ever hospitalization in two years^e^	28(12.2)	16(42.1)	5.25(2.47–11.17)	<0.001		

Abbreviation: MDR-TB, multidrug-resistant tuberculosis; COPD, chronic obstructive pulmonary disease; BMI, body mass index; GOLD, Global Initiative for Chronic Obstructive Lung Disease.

Note:

^a^ Excess alcohol consumption means more than 2 standard alcohol beverages per day.

^b^ TB contact was defined as a household member or colleague with TB.

^c^ In all patients post-bronchodilator forced expired volume in one second (FEV1)/forced vital capacity (FVC) was<0.7. Mild, FEV1 predicted<80%; moderate, 50%≤FEV1 predicted<80%; severe, 30% ≤FEV1 predicted<50%; very severe, FEV1 predicted≤30%.

^d^ Treatment duration of inhalation corticosteroids more than 2 months.

^e^Ever hospitalised in two years was due to an exacerbation of COPD.

## Discussion

MDR-TB is a form of TB caused by bacteria that do not respond to, at least, isoniazid and rifampicin, the two most powerful, first-line anti-TB drugs [5]. Our study showed that the proportion of patients with MDR-TB were significantly higher in pulmonary TB patients combined with COPD compared to those without COPD. Previous anti-TB treatment, migrant, cavity, and GOLD stage were the independent risk factors for MDR-TB among patients with COPD.

The available data on relationship between drug resistant TB and COPD are comparatively limited. A study of 309 civilians and 291 prisoners, reported a prevalence of isoniazid resistance of 60.2% and further suggested that this may be linked to an underlying diagnosis of COPD (P = 0.042) [17]. Another paper showed that COPD was not associated with the emergence of drug resistance on multivariate analysis by studying 276 TB cases (24 drug resistant cases) over a 6 year period. However, only 2 patients had drug resistant disease among 33 COPD patients in the study [18].

Accumulating evidences indicate that pulmonary TB can cause permanent obstructive or restrictive ventilation function impairment and change in lung anatomy, which contribute to developing COPD [8–10,19]. It is so widely recognized that previous anti-TB treatment is an established risk factor of MDR-TB [20–24]. COPD patients suffering from following conditions: smoking, impaired muco-ciliary clearance, long-term inhaled corticosteroids therapy, and sharing the significant genetic vulnerability component with TB, potentially increase the chance of active TB infection [11,12]. In our analysis, the proportion of patients with previous anti-TB treatment was significantly higher in patients with COPD. Moreover, there was a more TB reinfection in patients with COPD. It is not surprising that patients with relapse present higher rates of MDR-TB. In addition, previous study also indicated that CD4+ T lymphocytes were deplete in TB patients complicated with COPD and the patients with low levels of CD3, CD4 cell counts were susceptible to develop MDR-TB [25].

We noted that GOLD stage was an independent risk factors of being affected by MDR-TB in patients with COPD. The lower the value of FEV1 predicted is, the more obstructive the airflow is. The long-term damage to lung structures and respiratory function due to COPD further impairs the immunity of pulmonary [11,12]. It is possible that COPD patients with more severe airflow obstruction have more severe airway inflammation and decreased airway mucosal defense, predisposing them to increase the colonization or infection of the airway by drug resistant strains.

The prevalence of resistance to ofloxacin, MDR and MDR plus resistance to ofloxacin in patients with COPD were all higher than those without COPD. Previous study found that COPD was an independent predictor of fluoroquinolones resistance [13]. Because of favorable effects and slight adverse reaction, fluoroquinolones have been widely used for the treatment of bacterial infection in COPD patients, which could result in the development of resistance among these patients [13,26]. Fluoroquinolones are the core drugs for management of MDR-TB. However, the prevalence of MDR-TB with additional resistance to ofloxacin is significantly higher in COPD patients, which reduce the effect and options of treatment for MDR-TB [2,16]. Moreover, fluoroquinolones resistance among patients with MDR-TB has been regarded as an independent poor prognostic factor in previous reports [27,28]. MDR-TB in COPD patients will be more ominous.

In our study, some patients who experienced some alleviation of symptoms within the early phase of intensive therapy during hospitalization, interrupted further treatment after discharge. They were non-compliant in taking anti-TB drugs and returned to the clinic with recurrence of symptoms and radiographic progression of the disease. Inadequate treatment can select for the emergence of drug resistant mutations [20,22]. Therefore, during initiation of new case proper explanation and completion of the treatment is very important to avoid the development of future drug resistance in the society. Migrant are more likely to be indicated as poverty and having more limited access to medical treatment and healthcare services, and the crowded and poor living conditions may facilitate the spread of drug resistance strains. Cavity is a clinical manifestation in TB patients predicting a more severe illness. Consistent with previous literature [24,29–30], migrant and cavity are the risk factors for the development of MDR-TB.

The results of the study heighten the awareness of the health problem of TB combined with COPD and may have major policy implications for TB control. COPD is a major public health problem in China, accounting for 8.2% individuals aged ≥ 40 years [7]. Pulmonary TB patients with underlying COPD have the significance of harboring drug resistance, while patients are used to general hospitals or community clinics to see the doctor. China is the most populous country, with the hospital overcrowding and the inability to isolate resistant cases due to a lack of isolation facilities, which increase the nosocomial transmission of resistant strains. It should be of particular concern that the highly vulnerable individuals assemble in hospitals and other health-care settings.

Some limitations should be mentioned in our study. Firstly, as a hospital-based retrospective observational study, our data was limited by the relatively small numbers of MDR-TB and COPD patients, and reflective of clinical practice in 2011–2014. Furthermore, some of the heavy smoking patients who did not undergo spirometry might be suffering from uncharacterised, undiagnosed COPD. A larger sample and prospective study should be done to verify these associations. Secondly, in our study, the results of spirometry in COPD patients were recorded before TB diagnosis, thus the spirometry is the inability to reflect the lung function at the time of diagnosis of MDR-TB. However, in clinical practice, spirometry could be avoided in positive sputum culture patients owing to preventing the spread of TB. Thirdly, inhaled steroid therapy in COPD is a potential risk factor for the development of pneumonia. However, the association between inhaled steroid therapy and MDR-TB was not significant in this study, maybe due to missing data or an insufficient number of patients. The underlying mechanism association warrant further studies. Lastly, the role of antibiotics and systemic steroids therapy in COPD was not evaluated because the data of dosage and term prior to admission were not sufficiently robust due to a high percentage of unknown/unreliable results for self-reporting.

In conclusion, our study indicated that pulmonary TB patients complicated with COPD had a higher chance of developing MDR-TB. Clinicians should pay more attention to pulmonary TB patients with underlying COPD, especially those with being migrant, previous anti-TB therapy, cavity, and severe airway obstruction. Multi-faceted approaches will be necessary to reduce the emergency and transmission of MDR-TB in China, where most likely to be affected by the convergence of TB and COPD. Follow-up studies are needed to identify the causal mechanism of links between MDR-TB and COPD.
